# The Role of the Ikaros Family of Transcription Factors in Regulatory T cell Development and Function

**DOI:** 10.4172/2155-9899.1000495

**Published:** 2017-03-22

**Authors:** Amara Seng, Thomas M. Yankee

**Affiliations:** Department of Microbiology, Molecular Genetics, and Immunology, University of Kansas Medical Center, Kansas City, KS 66160, USA

**Keywords:** Regulatory T cell, Ikaros family, T cell development

## Abstract

Regulatory T (Treg) cells are a subset of immune cells that maintain homeostasis by promoting immune tolerance and suppressing the immune response via a variety of mechanisms such as secreting cytokines, killing reactive immune cells, and inducing anergy. Dysfunction of Treg cells has been implicated in inflammatory diseases such as autoimmunity and transplant rejection. Conversely, too many or hyperresponsive Treg cells has been observed in cancer and chronic infections. Treg cells have proven to be difficult to study as there are no definitive Treg surface markers. Additionally, Tregs can gain pro-inflammatory phenotype depending on stimuli. In this commentary, we discuss the expression and function of members of the Ikaros family of transcription factors during Treg cell development and activation.

## Introduction

Regulatory T (Treg) cells are approximately 3–5% of CD4^+^ T cells and function to promote immune tolerance and maintain immunologic homeostasis. Detailed mechanisms by which Treg cells down-regulate immune responses vary and have been reviewed elsewhere [1], but include secreting IL-10 and TGFβ, which suppress the proliferation and activation of pro-inflammatory conventional T (Tconv) cells [2]. Treg cells can also convert ATP into the immunosuppressive molecule adenosine and modulate metabolic activity [3]. Beyond their activities on other T cells, Treg cells can directly interact with dendritic cells and downregulate co-stimulation of Tconv cells [4]. Treg cells can also suppress macrophages and B cells [5–7].

Impaired Treg cell numbers or function has been linked to overactive immune responses, which contributes to diseases such as autoimmunity, allergy, and graft-versus-host disease [8–10]. Conversely, tumors often contain numerous Treg cells that suppress anti-tumor immunity [11]. Thus, the ability to manipulate the number or function of Treg cells would have profound therapeutic effects. For example, injection of Treg cells has been successfully used in murine models of multiple sclerosis, inflammatory bowel disease, and graft-versus-host disease [12–14]. However, translating these results into the clinic has been challenging, despite many attempts.

One challenge faced in Treg cell therapy has been to accurately identify this small cell population. Treg cells are characterized by high expression of the FOXP3 transcription factor. In addition, Treg cells express CD25, GITR, and CTLA-4, but low levels of CD127. In humans, these markers can also define activated T cells [15], making the isolation of a pure Treg population nearly impossible. Further, Treg cells can differentiate into pro-inflammatory CD4^+^ T cells under the appropriate conditions [16], so a pure Treg cell population could become a mixed population due to the plasticity of the differentiation state.

A potential solution to the problem of obtaining a pure, stable Treg cell population is to isolate Treg cells from the thymus instead of peripheral blood. Dijke et al. [14] showed that thymic-derived Treg cells were more effective than Treg cells obtained from peripheral blood in preventing graft-versus-host disease in a murine model of the disease. The most likely reason for the difference in efficacy is the stability of thymic Treg cell function, even in the presence of pro-inflammatory cytokines. These data demonstrate the value in identifying the characteristics that define thymic Treg cells and explain the stability of this population.

One defining feature of thymic Tregs is their high expression of Helios, a member of the Ikaros family transcription factors. In addition, Treg cells can express Ikaros, Aiolos, and Eos. Each Ikaros family member has four DNA-binding zinc finger motifs near the N-terminus and two C-terminal zinc fingers that mediate protein-protein interactions [17]. Each family member can homodimerize or heterodimerize via the C-terminal zinc fingers in every possible combination [17–21]. To further complicate this family of proteins, each member can undergo alternative splicing that eliminates one or more of the N-terminal zinc fingers [22–24]. Deletion of more than two zinc fingers results in a dominant negative form of the protein that can dimerize with other family members, but cannot bind DNA [17]. Examining the entire Ikaros family in Treg cells offers a tool analyze Treg cell development and function.

## The origin of Treg cell populations

**Figure 1:**
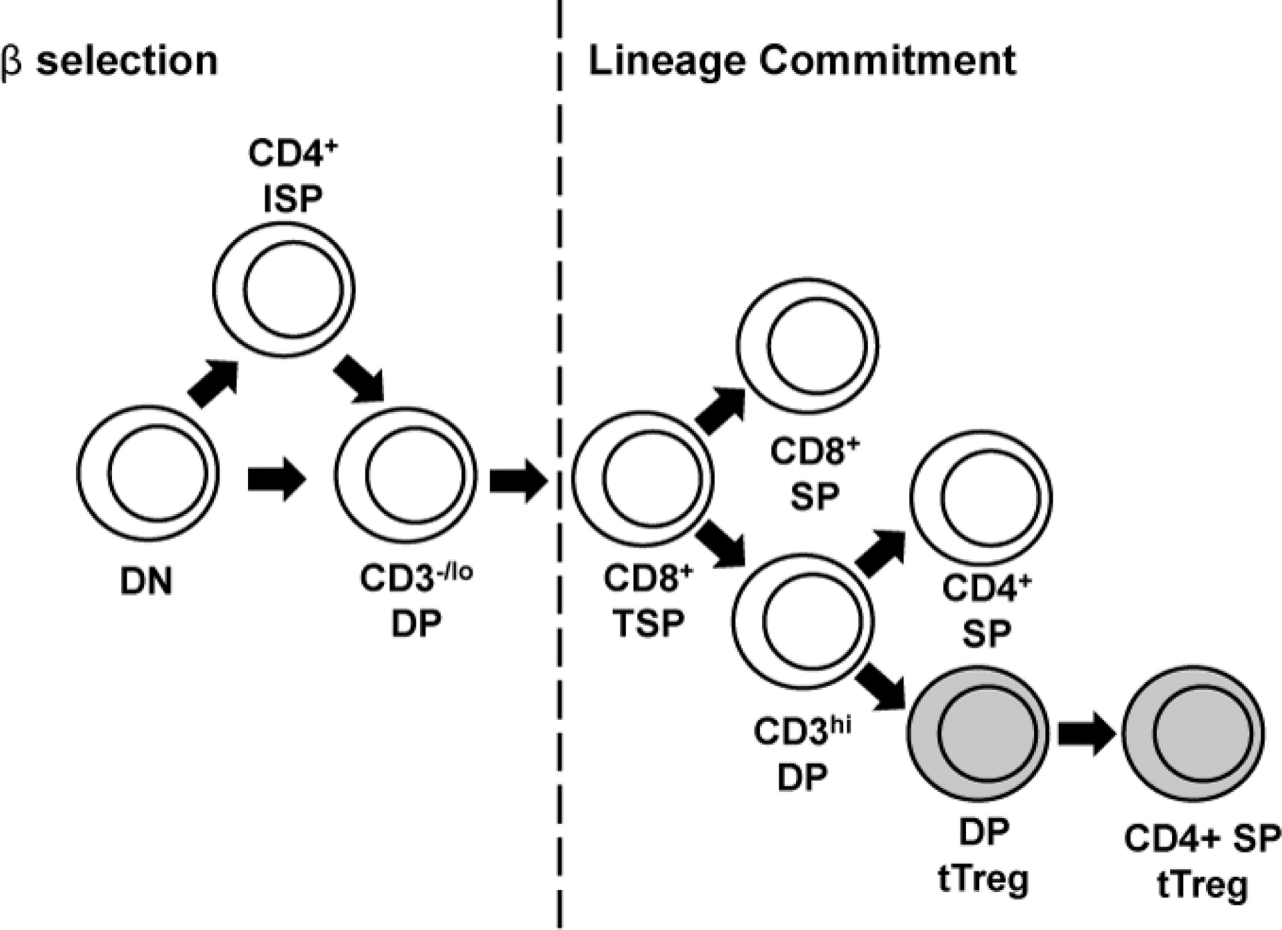
A model of Treg lineage commitment in T cell development. Tregs defined as CD25^hi^ FOXP3^hi^ Helios^+^ were identified in human thymus samples.

Peripheral blood contains a mixture of thymic-derived Treg (tTreg) and peripherally-derived Treg (pTreg) cells. Naïve CD4^+^ T cells can differentiate into pTreg cells in the presence of tolerogenic dendritic cells [25] while tTreg cells emerge from the thymus as a lineage of CD4^+^ T cells that is distinct from conventional CD4^+^ T cells (Figure 1). As yet, there are no accepted markers that can be used to distinguish tTreg from pTreg cells. However, an understanding of the developmental pathway leading to tTreg cells might lead to the identification of such markers.

In our two recent papers [26,27], we used multi-parameter flow cytometry to define novel subsets of human thymocytes with the goal of identifying the sites at which the major checkpoints occur. Consistent with previous reports [28–32], we found that CD4−CD8− double negative (DN) thymocytes differentiate into immature single positive (ISP) CD4^+^ thymocytes before expressing CD8 to become CD4^+^CD8^+^ double positive (DP) thymocytes [26]. However, this pattern only occurs in approximately half the subjects. In other individuals, CD4 and CD8 are expressed simultaneously, resulting in few ISP CD4^+^ cells. In rare cases, CD8 is expressed prior to CD4, resulting in an ISP CD8^+^ population. A major checkpoint that occurs during the DN to DP transition is expression of TCRβ. In some cells, this occurs during the ISP CD4^+^ stages while other cells do not express TCRβ until the early DP stages.

After β selection, DP thymocytes decrease their CD4 expression to become transitional single positive (TSP) CD8^+^ thymocytes (Figure 1) [27]. Positive selection occurs in the TSP CD8^+^ developmental stage. In addition, the initial steps towards CD4/CD8 lineage commitment begin in the TSP CD8^+^ stage. Cells destined to become mature CD8^+^ thymocytes continue to down-regulate CD4 and remain CD8^+^. Cells destined to become mature CD4^+^ thymocytes express CD4 to return to the CD4^+^CD8^+^ DP population before down-regulating CD8.

To identify the point of T cell development at which Treg cells emerge, we initially analyzed CD25 and FOXP3 expression. These markers were first detected in post-selection DP thymocytes that are newly committed to the CD4^+^ T cell lineage. Additionally, CD25^hi^FOXP3^hi^ cells were found in the mature CD4^+^ SP population.

## Using the Ikaros family to track Treg cell development

In addition to FOXP3, members of the Ikaros family of transcription factors are highly expressed in Treg cells [33–35]. During our analysis of human T cell development, we tracked the expression patterns of Ikaros family members. Using intracellular staining and flow cytometry, we showed that protein levels of Ikaros, Aiolos, and Helios increase when thymocytes undergo β selection, but the increase in Helios expression is greater than Ikaros and Aiolos. Further, the increase observed for Ikaros and Helios was transient while Aiolos levels remained elevated as thymocytes continued to mature. Similarly, the protein levels of Ikaros, Helios, and Aiolos increased when thymocytes underwent positive selection. Again, the spike in Ikaros and Helios expression was transient while Aiolos levels remained elevated in subsequent thymocyte populations [27].

These data indicate that the ratio of Ikaros family members changes at β selection and positive selection, suggesting that the nature of the dimers likely changes. The significance of changing the dimer composition as cells progress through T cell development is unknown, but is likely to influence the transcriptional activity of the complex [20].

Because all tTreg cells express Helios, we added the analysis of Helios expression to FOXP3 and CD25 to facilitate the identification of developing Treg cells [27]. Among mature CD3^hi^CD4^+^ thymocytes, Helios was only highly expressed in cells that also expressed FOXP3 and CD25. Helios^+^FOXP3^+^CD25^+^ thymocytes could also be found within the DP population, specifically among cells that had already undergone positive selection. These cells were between the TSP CD8^+^ thymocyte stage and the mature SP CD4^+^ stage, indicating that these cells are in the process of committing to the SP CD4^+^ lineage. Thus, using this combination of markers, we could trace to the developmental stages in which commitment to the CD4^+^ T cell lineage occurs. Independently of FOXP3 and CD25, Helios was also expressed in subsets of TSP CD8^+^ thymocytes. At this stage, Helios might mark the earliest Treg population or thymocytes undergoing negative selection [36].

## Ikaros family members in Treg cell subsets

Recently, there has been evidence that FOXP3^+^ Tregs can be divided into subsets based on gene expression and functionality [37]. For example, most Treg cells express Helios, including all thymic CD4^+^ Treg cells and approximately 70% of circulating Treg cells [33], and Treg cells that lack Helios express Aiolos [35]. Helios^+^ Treg cells can more effectively suppress cytokine production by Tconv cells [38,39], while Helios− Treg cells can secrete more pro-inflammatory cytokines such as IFNγ, IL-2, and IL-17 [35]. Production of these pro-inflammatory cytokines by Treg cells has been shown to be required for preventing experimental GvHD [40,41]. By contrast, Helios expression may be required for Treg-mediated suppression of T follicular helper (Tfh) cells and Type I helper T (Th1) cells [42]. Thus, analyzing expression of Ikaros family members in Tregs could aid in elucidating Treg function. Further, different populations of Treg cells might suppress different inflammatory diseases.

Besides differences in functionality between Helios^+^ and Helios^−^ Treg cells, Helios^+^ Treg cells are more stable under inflammatory conditions than Helios− Treg cells [14,33,43,44]. Knock-down of Helios in Treg cells decreased immunosuppressive function and survival *in vitro* and increased pathology in a murine colitis model [45]. Conversely, overexpression of Helios expression in Treg cells improved secretion of immunosuppressive cytokines and suppression of T cell proliferation *in vitro* [46].

Eos is also expressed in Treg cells and is often co-expressed with Helios [35]. Eos directly binds FOXP3 and is necessary for FOXP3-mediated gene repression [34,47]. Eos can also inhibit expression of non-Treg genes such as IL-2 [34,43,48]. Treg cells can convert into Tconv cells under inflammatory conditions and this process requires downregulation of Eos [43]. Additionally, knock-down of Eos in Treg cells results in reduced immunosuppressive activity both *in vitro* and *in vivo* in a murine colitis model [34].

These observations indicate that Ikaros family members can control the stability and phenotype of Treg cells and highlight the recent findings that there are multiple subsets of Treg cells. Different subpopulations of Treg cells may be required to suppress different diseases.

## Conclusion

The Ikaros family of transcription factors is a critically important factor in the development, stability, and function of Treg cells. We showed that analyzing the expression of Ikaros family members can be used to track Treg cell lineage commitment in the thymus. When studying the function of Ikaros family members in Treg cells, it is important to consider how expression of each family member impacts the other family members, as the composition of the Ikaros dimers will undoubtedly change when the expression of one family member is altered. This analysis is further complicated by the splice variants of each family member that might be expressed in each cell. In conclusion, understanding the role of the Ikaros family in Treg cells will further clarify the developmental pathway from which Treg cells originate, how Treg cells function, and how Treg cells might be used therapeutically.
